# Remarkably High-Performance Nanosheet GeSn Thin-Film Transistor

**DOI:** 10.3390/nano12020261

**Published:** 2022-01-14

**Authors:** Te Jui Yen, Albert Chin, Weng Kent Chan, Hsin-Yi Tiffany Chen, Vladimir Gritsenko

**Affiliations:** 1 Department of Electronics Engineering, National Yang Ming Chiao Tung University, Hsinchu 300, Taiwan; yenrick42269.ee05g@g2.nctu.edu.tw; 2 Department of Engineering and System Science, National Tsing Hua University, Hsinchu 300, Taiwan; kentchan00543@gapp.nthu.edu.tw (W.K.C.); hsinyi.tiffany.chen@gapp.nthu.edu.tw (H.-Y.T.C.); 3 Rzhanov Institute of Semiconductor Physics, Siberian Branch, Russian Academy of Sciences, 630090 Novosibirsk, Russia; grits@isp.nsc.ru; 4 Novosibirsk State University, 2 Pirogov Street, 630090 Novosibirsk, Russia; 5 Novosibirsk State Technical University, 20 Marks Avenue, 630073 Novosibirsk, Russia

**Keywords:** GeSn, nanosheet TFT, monolithic 3D IC, 3D brain-mimicking ICs

## Abstract

High-performance p-type thin-film transistors (pTFTs) are crucial for realizing low-power display-on-panel and monolithic three-dimensional integrated circuits. Unfortunately, it is difficult to achieve a high hole mobility of greater than 10 cm^2^/V·s, even for SnO TFTs with a unique single-hole band and a small hole effective mass. In this paper, we demonstrate a high-performance GeSn pTFT with a high field-effect hole mobility (μ_FE_), of 41.8 cm^2^/V·s; a sharp turn-on subthreshold slope (*SS*), of 311 mV/dec, for low-voltage operation; and a large on-current/off-current (I_ON_/I_OFF_) value, of 8.9 × 10^6^. This remarkably high I_ON_/I_OFF_ is achieved using an ultra-thin nanosheet GeSn, with a thickness of only 7 nm. Although an even higher hole mobility (103.8 cm^2^/V·s) was obtained with a thicker GeSn channel, the I_OFF_ increased rapidly and the poor I_ON_/I_OFF_ (75) was unsuitable for transistor applications. The high mobility is due to the small hole effective mass of GeSn, which is supported by first-principles electronic structure calculations.

## 1. Introduction

Thin-film transistors (TFTs) [1,2,3,4,5,6,7,8,9,10,11,12,13,14,15,16,17,18,19,20,21,22,23,24,25,26,27,28,29] have been investigated intensively in the past few decades [1,2,3] because of their ultra-low-energy-using process, usage of a small amount of material, and light transparency [4,5,6,7]. To realize system-on-panel (SoP) and monolithic three-dimensional (3D) integrated circuits (ICs) [8,9,10,11], high-performance n-type and p-type TFT devices (nTFT and pTFT, respectively) are required to form low-DC-power complementary TFTs (CTFTs) [12,13,14,15]. For oxide nTFTs, excellent device performance with a high field-effect mobility (μ_FE_), of ~100 cm^2^/V·s; a sharp turn-on subthreshold swing (*SS*), of ~100 mV/dec; and a large on-current/off-current (I_ON_/I_OFF_) ratio, of >10^6^, has been achieved using a SnO_2_ channel material [16,17,18,19,20]. However, because of the fundamental physical restrictions [30,31], the mobility of oxide pTFTs is generally less than 10 cm^2^/V·s [22,23,24,25], which remains a basic challenge for CTFTs. Although single-hole energy bands and small hole effective masses have been reported in metal-oxide SnO materials, the hole mobility of pTFTs is restrained by the requisite low-temperature process [22]. Alternatively, GeSn material also has a small hole effective mass and a direct energy bandgap [32,33,34]. In this paper, we report poly-GeSn pTFT with a high *μ_FE_* (41.8 cm^2^/V·s), a sharp SS (311 mV/dec), and a large I_ON_/I_OFF_ value (8.9 × 10^6^). Although an even larger hole mobility, of 103.8 cm^2^/V·s, is obtained in a thicker GeSn channel, there is a tradeoff with a poor I_OFF_, with an I_ON_/I_OFF_ of only 75. The crucially large I_ON_/I_OFF_ was achieved using an ultra-thin (7 nm) nanosheet GeSn. The I_OFF_ leakage is the crucial issue for highly scaled 3 and 2 nm node silicon (Si) transistors. To decrease the I_OFF_, an ultra-thin (7 nm) channel layer is used for Si nanosheet FETs on 12-inch wafers. It is important to note that although many papers have reported the device performance using the monolayer two-dimensional (2D) materials, there is no manufacture solution for a 12-inch Si wafer till date. X-ray photoelectron spectroscopy (XPS) analysis revealed that the Ge/Sn ratio in the GeSn film was 7. First-principles electronic structure calculations show that the high mobility is due to the smaller hole effective mass of Ge_0.875_Sn_0.125_, which is lower than that of Ge. The low fabrication temperature (350 °C) and a high-performance nanosheet GeSn TFT are an enabling technology for SoP, monolithic 3D ICs, and 3D brain-mimicking ICs.

## 2. Materials and Methods

A 500 nm thick SiO_2_ layer was formed on a p-type Si wafer to mimic a glass substrate. Subsequently, 50 nm of TaN was deposited by a reactive sputtering system and served as the gate electrode. Then, the gate insulator was deposited with a 40 nm thick high-dielectric constant (high-κ) HfO_2_ layer and a 2 nm SiO_2_ interfacial layer. The gate insulator was subjected to 350 °C post-deposition annealing in ambient O_2_ for 30 min. Thereafter, GeSn layers with a thickness of 5, 7, or 9 nm were deposited by sputtering Ge and Sn targets at 80 and 10 W, respectively, under an Ar gas flow of 24 sccm. Next, the GeSn layer was annealed at 350 °C for 30 s by rapid thermal annealing in N_2_ ambient. Finally, 30 nm of Ni was deposited and patterned as the source and drain electrodes to form the TFTs. The length and width of the bottom-gate GeSn pTFTs were 50 and 500 μm, respectively. The electrical characteristics were measured using an HP 4155 B parameter analyzer and a probe station. All the devices were measured at 25 °C, the room temperature in a lab environment. The GeSn channel layer was analyzed by X-ray photoelectron spectroscopy (XPS, Thermo Nexsa, MA, USA). The device structure was examined using high-resolution transmission electron microscopy (TEM, FEI Talos F200X, OR, USA). The crystallinity of the GeSn layer was measured by X-ray diffraction (XRD) using a Bede D1 high-resolution XRD analyzer (Durham, England). First-principles electronic structure calculations were carried out using the Vienna ab initio simulation package (VASP) [35] and aimed to disclose the electronic structure of Ge_0.875_Sn_0.125_. The projector augmented wave (PAW) approach was applied to describe the interactions between the core electrons and nuclei [36,37]. The valence electrons explicitly treated were (4s^2^, 4p^2^) and (5s^2^, 5p^2^) for Ge and Sn, respectively. The exchange correlation of electrons was described using Heyd–Scuseria –Ernzerhof (HSE) hybrid functionals [38]. The self-consistent calculation converged at 10^−6^ eV. The structures were optimized using a conjugated-gradient algorithm until the ionic forces were smaller than 0.0001 eV/Å with a plane wave cutoff of 400 eV, and the corresponding k-point mesh of 5 × 5 × 5 was applied to the optimized structure of the diamond cubic ${\mathrm{Ge}}_{0.875}{\mathrm{Sn}}_{0.125}$ model with a lattice constant of 5.763 Å, containing eight atoms (Figure S1). Density of state (DOS) calculations were performed using a denser k-point mesh, of 6 × 6 × 6. The SUMO Python Package [39,40] and Vaspkit code [41] were employed to generate symmetry K-Path for the band structure calculation and for post processing of the effective mass extraction from the band.

## 3. Results

**Figure 1:**
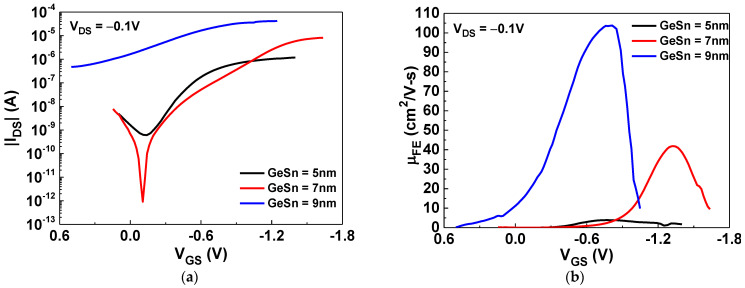
The (**a**) |*I_DS_*|–*V_GS_* and (**b**) *μ_FE_*–*V_GS_* characteristics of the GeSn/SiO_2_/HfO_2_ pTFTs with different channel thicknesses.

Figure 1a shows the drain-source current versus the gate-source voltage (*I_DS_-V_GS_*) characteristics of GeSn/SiO_2_/HfO_2_ pTFTs with GeSn thicknesses of 5, 7, or 9 nm. The device with a channel thickness of 7 nm exhibited the best performance, with an I_ON_/I_OFF_ value of 8.9 × 10^6^. The gate-source current versus the gate-source voltage (|*I_GS_*|−*V_GS_*) of the TFT devices with different GeSn film thicknesses is displayed in Figure S2. Figure 1b displays the field-effect mobility versus the gate-source voltage (*μ_FE_-V_GS_*) characteristics of the GeSn pTFTs, which were measured under a small *V_DS_* (−0.1 V). Here, the hole mobility values increase with the GeSn layer thickness, which is consistent with the increasing trend of I_ON_. This is due to the decreased depletion width of GeSn by the gate and surface potential, which provides more carriers to transport from the source to the drain. The peak mobilities of the GeSn TFTs with GeSn thicknesses of 5, 7, and 9 nm were 3.9, 41.8, and 103.8 cm^2^/V·s, respectively. Although the device with a GeSn thickness of 9 nm showed the highest mobility, the poor SS (1560 mV/dec) and an I_ON_/I_OFF_ of only 75 make it unsuitable for device applications. The thin (5 nm) channel thickness exhibited 10 times lower mobility than the 7 nm GeSn device, which is attributed to the lack of carriers and strong interfacial scattering [26,27]. The 7 nm GeSn TFT device showed a large I_ON_/I_OFF_, of 8.9 × 10^6^; a good SS value, of 311 mV/dec; and a high *μ_FE_*, of 41.8 cm^2^/V·s, which is much better than those of traditional oxide pTFTs and shows the high potential for future SoP and monolithic brain-mimicking IC applications. In addition, such a high hole mobility is similar to that of the single-crystal Si used for standard ICs [42]. It is important to note that the nanosheet GeSn thickness of 7 nm is exactly the same as that of the single-crystal Si nanosheet FET used for 2 nm node technology manufacture.

**Figure 2:**
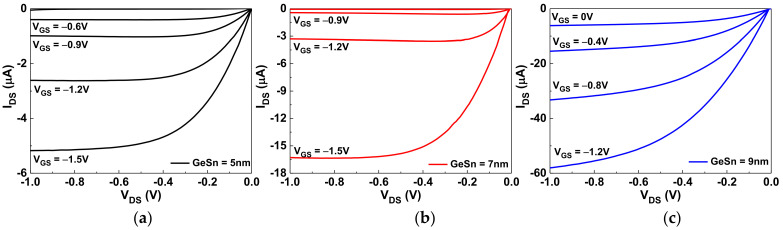
The *I_DS_*–*V_DS_* characteristics of the GeSn/SiO_2_/HfO_2_ pTFTs with (**a**) 5 nm, (**b**) 7 nm, and (**c**) 9 nm channel thicknesses.

The drain-source current versus the drain-source voltage (*I_DS_-V_DS_*) characteristics of GeSn pTFT devices with GeSn thicknesses of 5, 7, or 9 nm are shown in Figure 2a–c, respectively. The saturation *I_DS_* increases with increasing GeSn channel thickness, and a higher *I_DS_* leads to a higher *μ_FE_*, as shown in Figure 1b. The *I_DS_*–*V_DS_* curves for 5 and 7 nm thicknesses of GeSn display good I_DS_ saturation characteristics. In contrast, the 9 nm thick GeSn device shows poor saturation characteristics, which is due to excessive carrier conduction and poor channel pinch-off. In addition, the non-negligible I_DS_ at V_GS_ = 0 V will lead to high standby power.

**Figure 3:**
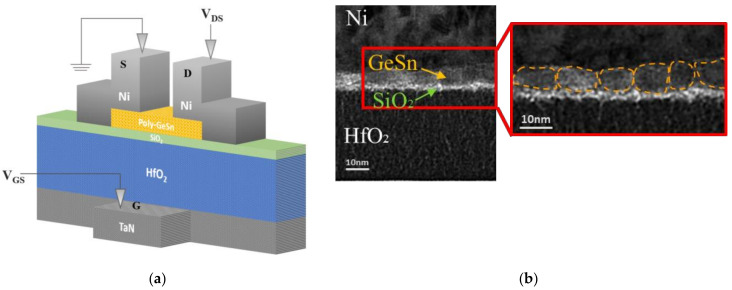
(**a**) The schematic device diagram and (**b**) the cross-sectional TEM image of a bottom-gate GeSn pTFT.

Figure 3a illustrates the schematic device structure diagram of the bottom-gate GeSn pTFT. High-work-function Ni was formed on GeSn and used as the drain and source electrodes. The HfO_2_ and thin SiO_2_ stack served as the gate dielectric, in which SiO_2_ was used to minimize the remote phonon scattering effects from high-κ HfO_2_ [17,20,43,44,45]. In this study, a high-κ gate dielectric was used to increase the gate capacitance and I_ON_, which is widely used for Si metal-oxide-semiconductor (MOS) FET and TFT devices. The device structure was further verified using cross-sectional TEM. As depicted in Figure 3b, the thicknesses of the GeSn channel layer and the SiO_2_ interfacial layer on high-k HfO_2_ are 7 and 2 nm, respectively. Here, the crystal grains in the TEM image are marked with a yellow dashed line. Figure S3a and Figure S3b show the TEM images of the device with GeSn layers annealed at 300 and 350 °C, respectively. A 2 nm SiO_2_ layer is added between HfO_2_ and GeSn. Via an atomic force microscope (AFM), Figure S4 exhibits the surface roughness of the GeSn layer annealed at different temperatures. The surface roughness of the GeSn layer degrades with increasing annealing temperature. The device annealed at 300 °C displays the best surface smoothness and uniformity (Figure S4a); however, the hole *μ_FE_* is only 3.71 cm^2^/V·s, as depicted in Figure S5. The crystalline size depends on the GeSn thickness and annealing temperature. However, the increasing GeSn thickness increases the device I_OFF_ leakage. The increasing annealing temperature degrades mobility by increasing surface roughness (Figure S4c). Therefore, there is a tradeoff between the channel layer surface roughness, uniformity, and carrier mobility. The 350 °C annealing is the best condition to increase μ*_FE_* of the nanosheet FET. Further hole *μ_FE_* and I_OFF_ tradeoff may be possible by using a 6 nm GeSn layer.

**Figure 4:**
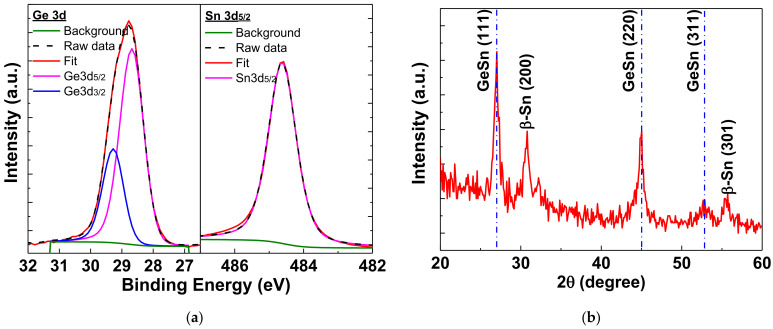
The (**a**) XPS spectra of Ge 3d and Sn 3d_5/2_ and (**b**) XRD analyses of GeSn film annealed at 350 °C.

XPS analysis was conducted to examine the composition of the GeSn layer. Through XPS measurements, as depicted in Figure S6, the ratio of Ge/Sn was determined to be 7. Figure 4a shows the Ge 3d and Sn 3d_5/2_ core spectra. There is no oxide compound signal of GeO_x_ or SnO_x_ in the Ge 3d and Sn3d_5/2_ spectra [46], which is one of the reasons for the high mobility. As depicted in Figure 4b, the XRD analysis reveals that the GeSn layer is polycrystalline after annealing at 350 °C. The diffraction peaks related to GeSn are crystal orientations GeSn (111), GeSn (220), and GeSn (311), corresponding to 2θ values of 27.0°, 45.0°, and 52.7°, respectively, which are similar to previously published data [29,47,48,49]. Additionally, at 2θ = 30.79° and 55.03°, diffraction peaks of β-Sn (200) and β-Sn (301) were observed [22,49,50], which could be the Sn precipitated during the rapid thermal annealing at 350 °C. 

**Figure 5:**
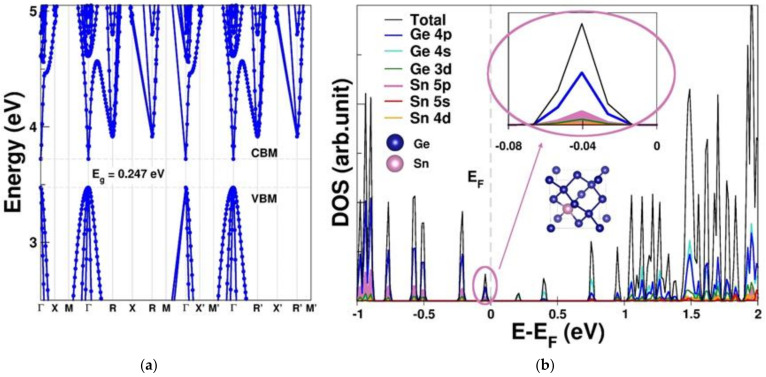
(**a**) The band structure and (**b**) the total density of states (DOS), the orbital-decomposed DOS, the projected DOS of Sn, and the local DOS near the VBM (insert) of Ge_0.875_Sn_0.125_.

To thoroughly comprehend the fundamental physical properties related to the hole mobility of the GeSn pTFT, the electronic structure and hole effective mass were computed based on first principles. Figure 5a,b present the band structure and density of states (DOS) of Ge_0.875_Sn_0.125_, respectively, revealing a direct bandgap of 0.25 eV. Our calculated outcome is analogous to that of previous work, where the bandgap of GeSn is expected to turn directly when the Sn/Ge ratio is larger than 8% [33]. The contributions of each orbital in the valence band maximum (VBM) of ${\mathrm{Ge}}_{0.875}{\mathrm{Sn}}_{0.125}$ were further investigated using the total DOS, the orbital-decomposed DOS, the projected DOS of Sn, and the local DOS near the VBM, as shown in Figure 5b. These results lead to the following conclusions: (1) Both the valence band and the conduction band are primarily contributed by the Ge orbitals (Figure S7). (2) Figure 5b shows that the topmost valence band is predominantly contributed by both Ge 4p and Sn 5p orbitals, reinforcing the electron density in the vicinity of the VBM. (3) In contrast, for the region near and beyond the conduction band minimum (CBM), the major contribution is from all Ge orbitals (4s > 4p > 3d), but not the Sn orbitals, supporting the predominant contribution of Sn orbitals to the VBM. In addition, the calculated effective mass and the energy bandgap of Ge_0.875_Sn_0.125_ from Figure 5a are summarized in Table 1. The heavy-hole effective mass at the Γ point ($m_{hh}^{*\Gamma }$) is −0.225 $m_{0}$ (the unit of free electron mass), while the light-hole effective mass at the Γ point ($m_{lh}^{*\Gamma }$) is −0.028 $m_{0}$, indicating that the heavy-hole effective mass dominates the total transport mass. Table 1 also shows that the $m_{hh}^{*\Gamma }$ of Ge_0.875_Sn_0.125_ is less than that (0.28 *m*_0_) of Ge and half that of Si (0.49 *m*_0_) [51]. This is the reason why GeSn has been proposed for pMOS or pTFT. However, the reported GeSn pTFTs in the literature suffered from poor I_ON_/I_OFF_ [26,27,28,29], which is due to the leakage current of the small energy bandgap. 

Table 2 displays the crucial TFT device parameters of various poly-GeSn TFTs [26,27,28,29]. The remarkably high I_ON_/I_OFF_ and relatively sharp SS are the advantages of this study. The excellent device performance is due to the ultra-thin nanosheet GeSn layer, with a thickness of only 7 nm. Although the mobility can reach higher than 100 cm^2^/V·s with a thickness of 9 nm, the SS and I_ON_/I_OFF_ values are degraded and unfavorable for transistor applications. The low process temperature (350 °C) and high-performance nanosheet GeSn pTFT in this work are promising for SoP and monolithic 3D IC applications.

## 4. Conclusions

In this study, a high-performance poly-GeSn pTFT was achieved with an excellent transistor performance (41.8 cm^2^/V·s), a sharp SS (0.31 V/dec), and a large I_ON_/I_OFF_ value (8.9 × 10^6^). This was achieved using an ultra-thin (7 nm) nanosheet GeSn layer that can be depleted by gate and surface potentials. The high hole mobility is related to its small heavy-hole effective mass, of 0.225*m*_0_. The remarkably high performance and low thermal budget of the nanosheet GeSn pTFT are an enabling technology for CTFTs, SoP, monolithic 3D ICs, and 3D brain-mimicking ICs.

## Figures and Tables

**Table 1:** Comparison of effective masses at gamma of heavy-hole ($m_{hh}^{*\Gamma },\;{\mathrm{in}\;m}_{0}$) and light-hole ($m_{lh}^{*\Gamma },\;{\mathrm{in}\;m}_{0}$) direct and indirect bandgaps ($E_{g}{}_{\mathrm{direct}}$ and $E_{g}{}_{\mathrm{indirect}}\;\mathrm{in}\;\mathrm{eV}$) of Ge_0.875_Sn_0.125_, Ge [51], and Si [51].

**Reference**	Material	$m_{hh}^{*\Gamma }\left(m_{0}\right)$	$m_{lh}^{*\Gamma }\;(m_{0})$	$E_{g}{}_{direct}\;(\mathrm{eV})$	$E_{g}{}_{indirect}\;(\mathrm{eV})$
This work	Ge_0.875_Sn_0.125_	0.225	0.028	0.25	--
52 work	Ge	0.28	0.044	--	0.66
52	Si	0.49	0.16	--	1.12

**Table 2:** Comparison of several important parameters among various poly-GeSn TFT devices.

Reference	Poly-GeSn Thickness (nm)	Highest Process Temperature (°C)	*μ_FE_* (cm^2^/V·s) @V*_DS_* (V)	SS (V/Decade)	I_ON_/I_OFF_
26	10	300	54 @ −0.5	--	1.2 × 10^2^
27	-- (nanowire)	440	14.54 @ −0.2	1.87	5.3 × 10^3^
28	12	500	39.3 @ −0.05	--	1.7 × 10^4^
29	15	500	20 @ −0.05	1	1.1 × 10^4^
This work	9	350	103.8 @ −0.1	1.56	75
This work	7	350	41.8 @ −0.1	0.31	8.9 × 10^6^

## Data Availability

The data presented in this study are available on request from the corresponding author. The data are not publicly available due to privacy.

## Supplementary Materials

The following are available online at https://www.mdpi.com/article/10.3390/nano12020261/s1, Figure S1: Optimized structure of the diamond cubic ${\mathrm{Ge}}_{0.875}{\mathrm{Sn}}_{0.125}$ model with the lattice constant of 5.763 Å, containing eight atoms by first-principles calculations, Figure S2: The |*I_GS_*|−*V_GS_* characteristics of the GeSn/SiO_2_/HfO_2_ pTFTs with different channel thickness, Figure S3: The cross-sectional TEM image of GeSn pTFT with GeSn annealed at (a) 300 and (b) 350 °C, Figure S4: The surface roughness of GeSn films annealed at (a) 300 °C, (b) 350 °C and (c) 400 °C measured by AFM, Figure S5: The |*I_DS_*|−*V_GS_* and *μ_FE_*−*V_GS_* characteristics of the GeSn pTFTs with 7 nm channel thickness and annealed at 300 °C, Figure S6: The XPS spectrum of ${\mathrm{Ge}}_{0.875}{\mathrm{Sn}}_{0.125}$, Figure S7: Total density of state (DOS) and projected DOS of Ge and Sn of ${\mathrm{Ge}}_{0.875}{\mathrm{Sn}}_{0.125}$ by first-principles calculations.
