# Supplementary material for: Remarkably High-Performance Nanosheet GeSn Thin-Film Transistor
Source: Nanomaterials (Basel). 2022 Jan 14;12(2):261. doi: 10.3390/nano12020261 (PMC8777649; doi:10.3390/nano12020261)
Supplement: Supplementary file 1 [file nanomaterials-12-00261-s001.zip › nanomaterials-1529701-supplementary.pdf]

nanomaterials

Supplementary Material

Remarkably High-Performance Nanosheet GeSn Thin-Film Transistor

Te Jui Yen [1], Albert Chin [1,*], Weng Kent Chan [2], Hsin-Yi Tiffany Chen [2] and Vladimir Gritsenko [3,4,5]

[1] Department of Electronics Engineering, National Yang Ming Chiao Tung University, Hsinchu 300, Taiwan; yenrick42269.ee05g@g2.nctu.edu.tw
[2] Department of Engineering and System Science, National Tsing Hua University, Hsinchu 300, Taiwan; kentchan00543@gapp.nthu.edu.tw (W.K.C.); hsinyi.tiffany.chen@gapp.nthu.edu.tw (H.-Y.T.C.)
[3] Rzhanov Institute of Semiconductor Physics, Siberian Branch, Russian Academy of Sciences, 630090 Novosibirsk, Russia; grits@isp.nsc.ru
[4] Novosibirsk State University, 2 Pirogov Street, 630090 Novosobirsk, Russia
[5] Novosibirsk State Technical University, 20 Marks Avenue, 630073 Novosibirsk, Russia
* Correspondence: achin@nycu.edu.tw; Tel.: +886-3-5731841

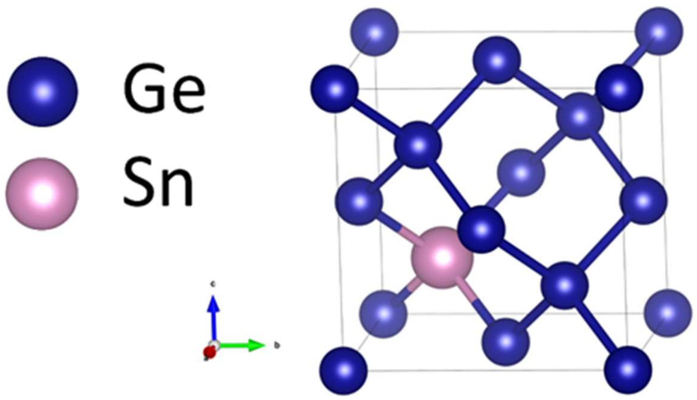

Figure S1. Optimized structure of the diamond cubic $Ge_{0.875}Sn_{0.125}$ model with the lattice constant of 5.763 Å, containing eight atoms by first-principles calculations.

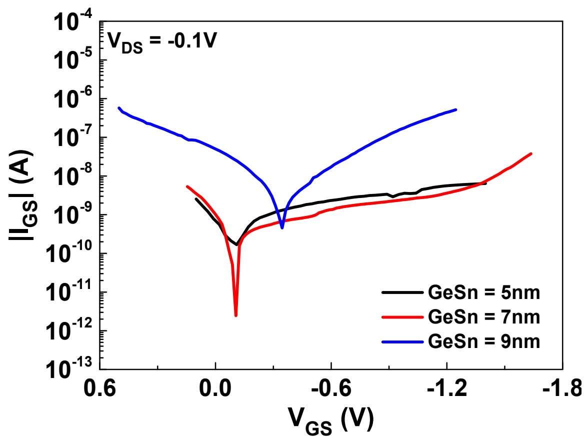

Figure S2. The $|I_{GS}|-V_{GS}$ characteristics of the GeSn/SiO_2/HfO_2 pTFTs with different channel thickness.

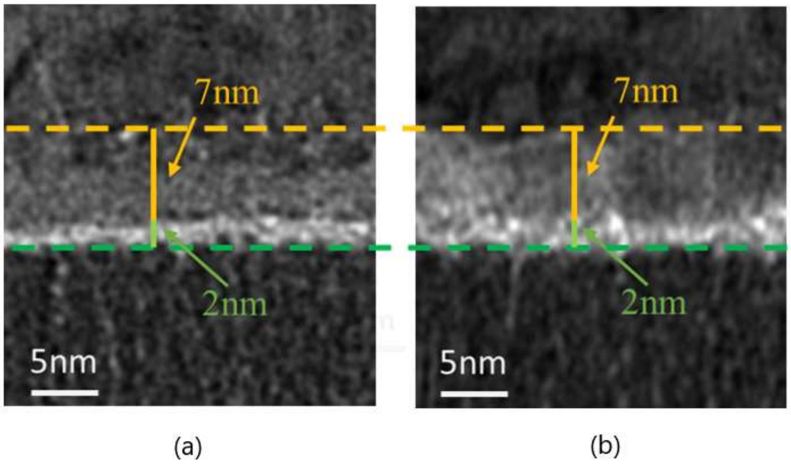

Figure S3. The cross-sectional TEM image of GeSn pTFT with GeSn annealed at (**a**) 300 and (**b**) 350 °C.

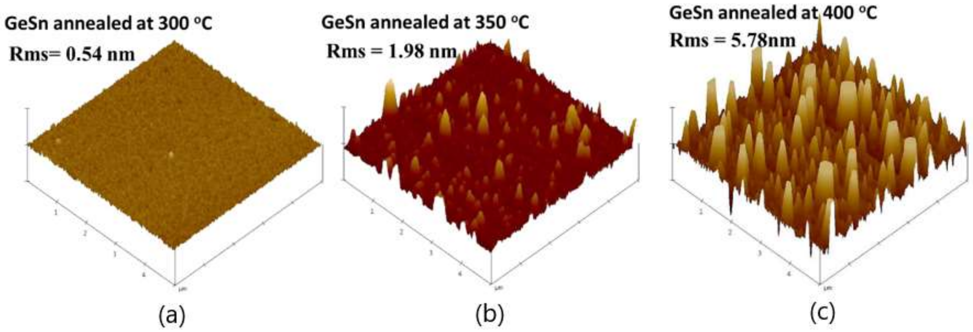

Figure S4. The surface roughness of GeSn films annealed at (**a**) 300 °C, (**b**) 350 °C and (**c**) 400 °C measured by AFM.

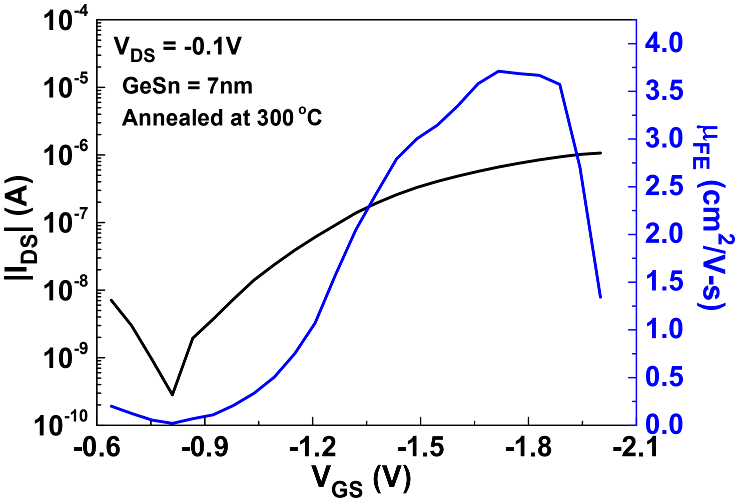

Figure S5. The $|I_{DS}|$–V_{GS} and μ_{FE}-V_{GS} characteristics of the GeSn pTFTs with 7nm channel thickness and annealed at 300 °C.

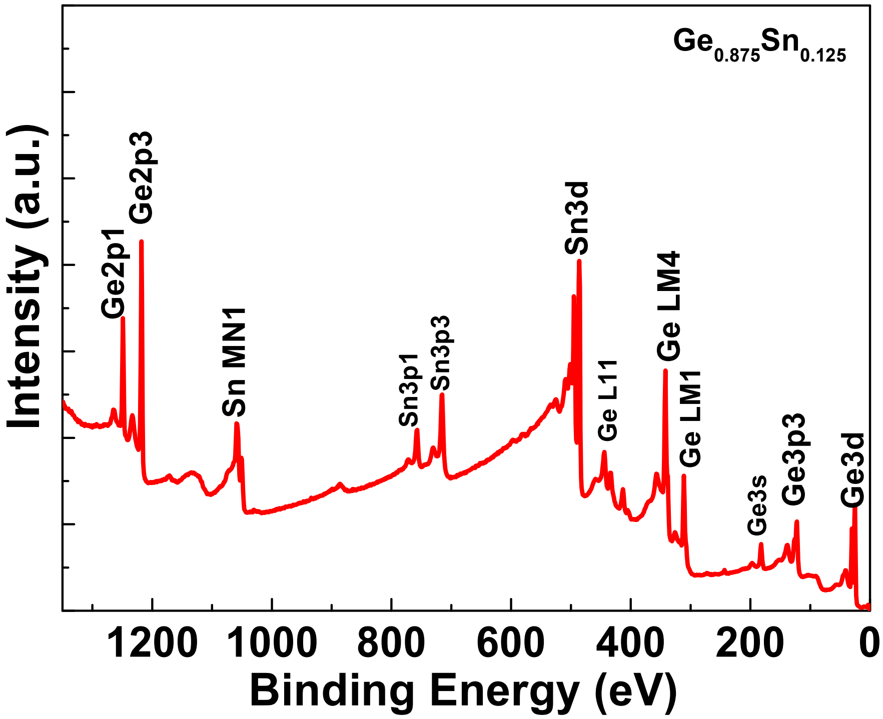

Figure S6. The XPS spectrum of $Ge_{0.875}Sn_{0.125}$.

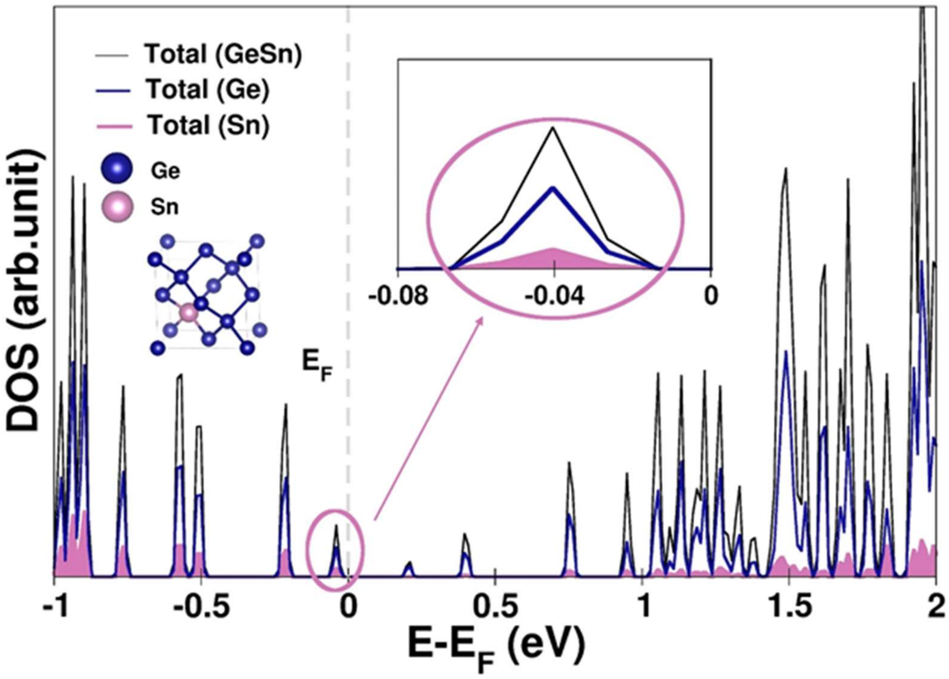

Figure S7. Total density of state (DOS) and projected DOS of Ge and Sn of $Ge_{0.875}Sn_{0.125}$ by first-principles calculations.
